# Supplementary figures and images for: Reprogramming LCLs to iPSCs Results in Recovery of Donor-Specific Gene Expression Signature
Source: PLoS Genet. 2015 May 7;11(5):e1005216. doi: 10.1371/journal.pgen.1005216 (PMC4423863; doi:10.1371/journal.pgen.1005216)

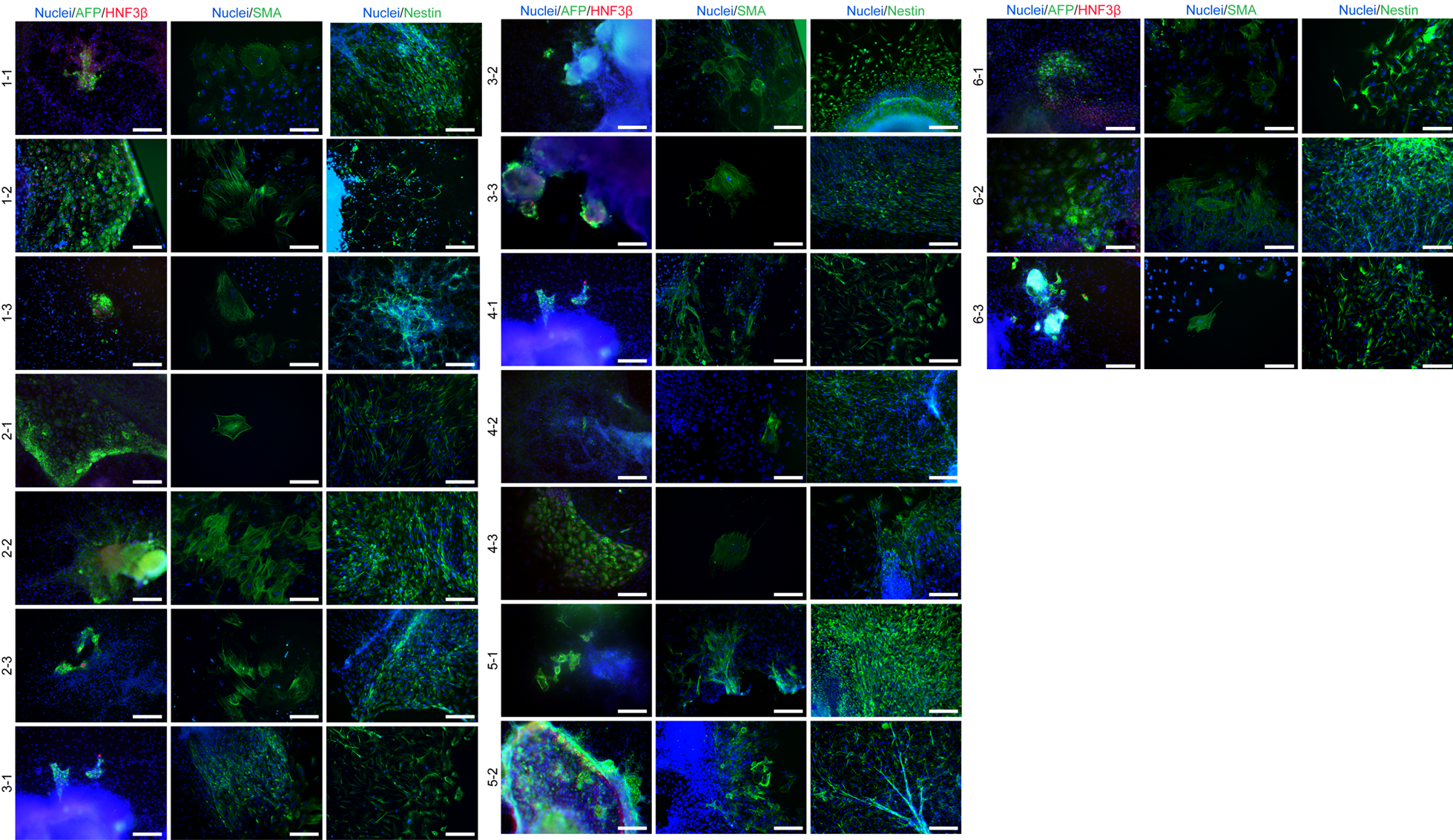

Supplement: S1 Fig — Immunocytochemistry approach to test for a cell line’s ability to spontaneously differentiate through endoderm: HNF3β and α-fetoprotein (AFP), mesoderm: smooth muscle actin (SMA), and ectoderm: nestin lineages. Scale bar: 200 μm. Individual channel levels, brightness, and contrast were adjusted using Adobe Photoshop CS6. (TIF) [file pgen.1005216.s001.tif]

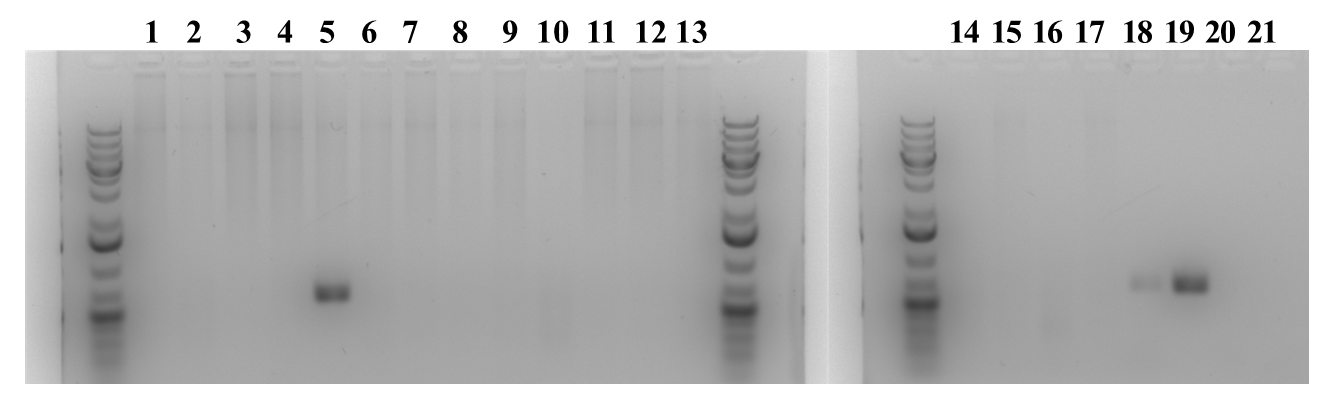

Supplement: S2 Fig — Reprogramming vectors and Epstein-Barr virus are absent in all iPSC lines except 3–2 (see S3 Fig)1.4–1 iPSC, 2. 6–3 iPSC, 3. 5–2 iPSC, 4. 3–3 iPSC, 5. 3–2 iPSC, 6. 6–2 iPSC, 7. 2–3 iPSC, 8. 5–1 iPSC, 9. 1–2 iPSC, 10. 1–1 iPSC, 11. 3–1 iPSC, 12. 3–3 iPSC, 13. 2–2 iPSC, 14. 2–1 iPSC, 15. 4–2 iPSC, 16. 1–3 iPSC, 17. 6–1 iPSC, 18. Reprogramming plasmids (positive control) 19. LCL DNA (YRI lines 18508 and 19238, positive control). 20. Fibroblast DNA (negative control), 21. Water. (TIF) [file pgen.1005216.s002.tif]

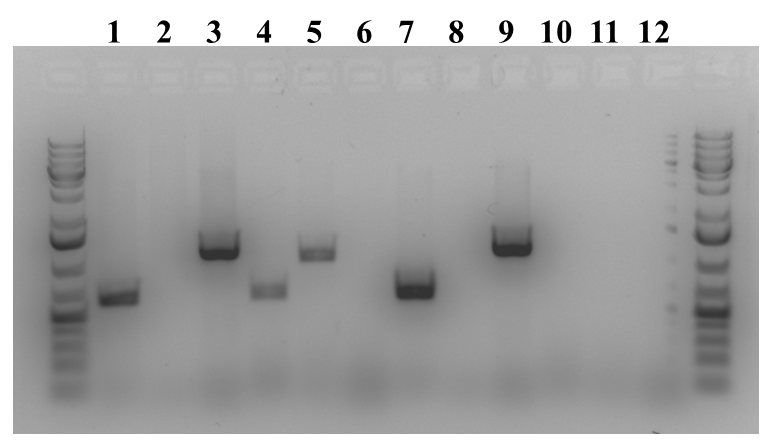

Supplement: S3 Fig — iPSC 3–2 exhibits presence of EBV and absence of reprogramming plasmids.1.3–2 iPSC/EBNA-1 primer set, 2. 3–2 iPSC/PXCLE primer set, 3. 3–2 iPSC/EBV primer set, 4. Reprogramming plasmid template/EBNA-1 primer set, 5.Reprogramming plasmid template/PXCLE primer set, 6. Reprogramming plasmid template/EBV primer set, 7. LCL DNA/EBNA-1 primer set, 8. LCL DNA/PCXLE primer set, 9. LCL DNA/EBV primer set. 10. Fibroblast DNA/EBNA-1 primer set 11. Fibroblast DNA/PCXLE primer set 12. Fibroblast DNA/EBV primer set. (TIF) [file pgen.1005216.s003.tif]

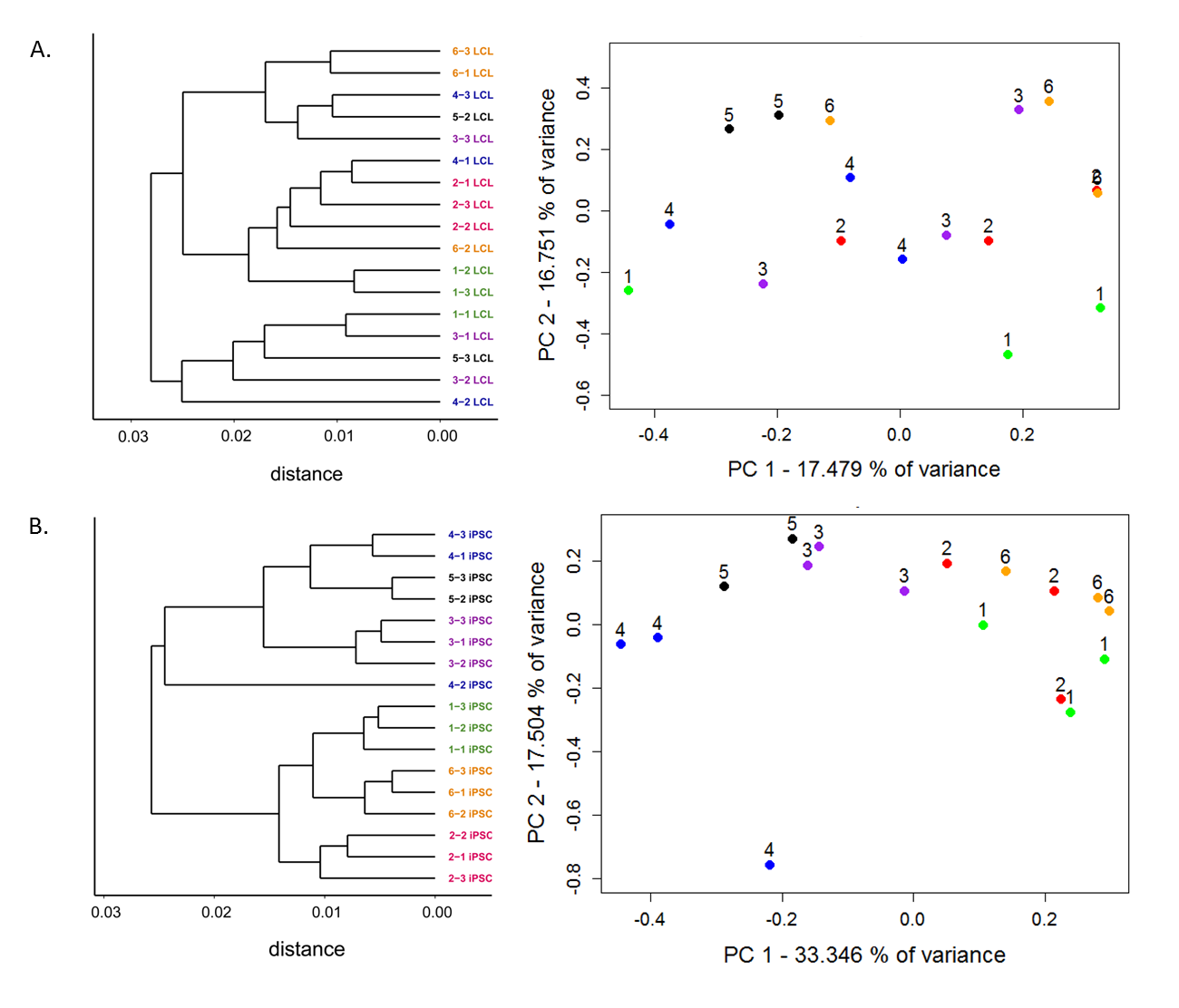

Supplement: S4 Fig — A. Results from hierarchical clustering analysis of microarray gene expression and expression data projections on principal components axes 1 and 2 from cycle 7 LCLs and B. iPSCs. Includes data from all lines. (TIF) [file pgen.1005216.s004.tif]

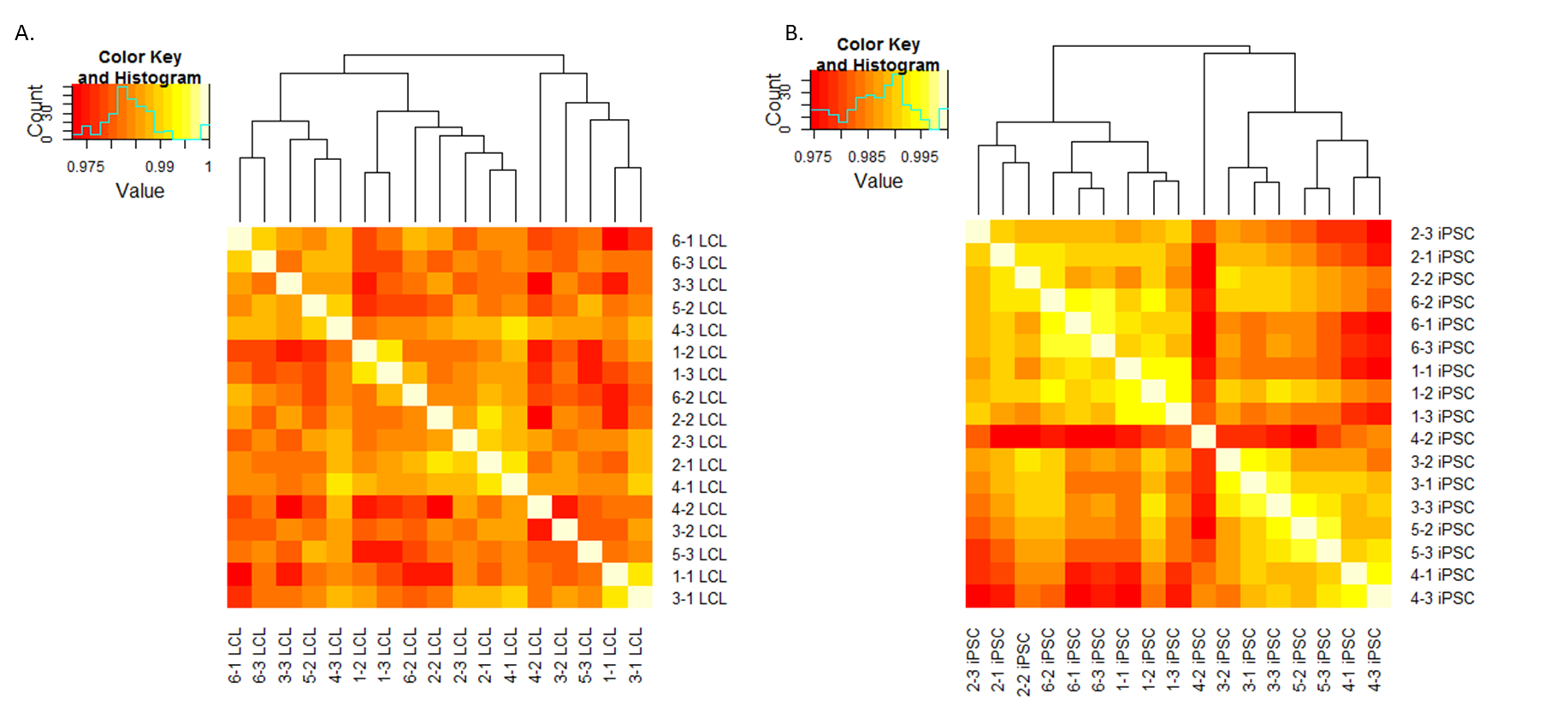

Supplement: S5 Fig — Heatmap generated from pairwise correlation matrix (pearson product-moment correlation coefficients) for A. LCLs and B. iPSCs. Includes data from all lines. (TIF) [file pgen.1005216.s005.tif]

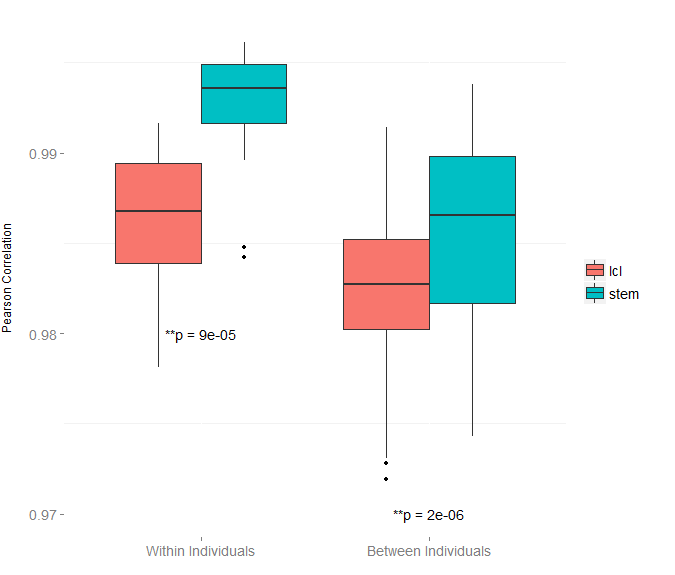

Supplement: S6 Fig — Pairwise Pearson correlation coefficients for gene expression data from lines derived from the same individual and across different individuals for both cell types. iPSCs demonstrate increased correlation both within and across individuals compared with LCLs. Includes data from all lines. (TIFF) [file pgen.1005216.s006.tiff]

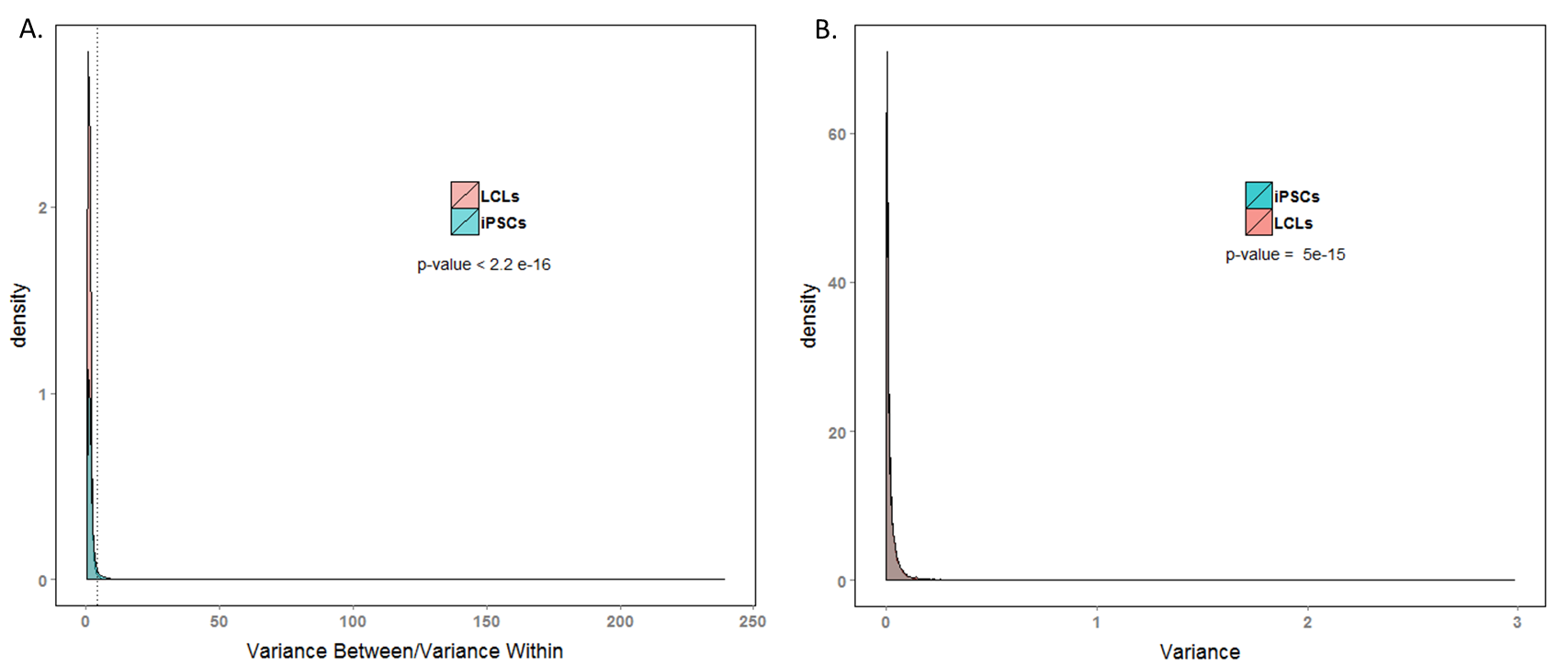

Supplement: S7 Fig — A. Density plot of between donor variance to within donor variance in gene expression for all expressed genes in iPSCs and LCLs The dotted line indicates the threshold corresponding with significant association between gene expression and individual of origin. All data are plotted; calculations exclude the outlier. B. Density plot of total variance in LCLs and iPSCs. All data are plotted; calculations exclude the outlier. (TIF) [file pgen.1005216.s007.tif]

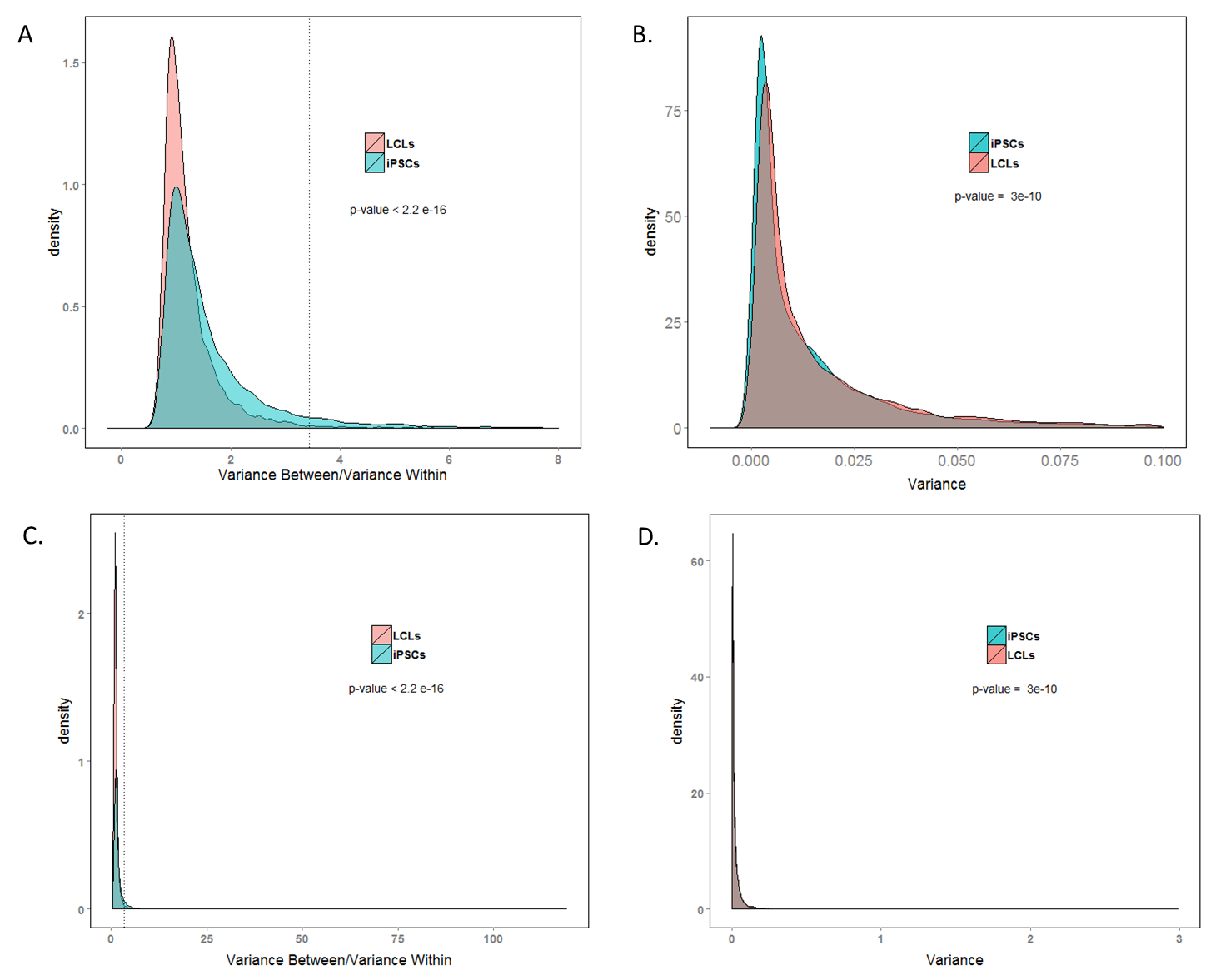

Supplement: S8 Fig — A. Density plot of between donor variance to within donor variance in gene expression for all expressed genes in iPSCs and LCLs. The dotted line indicates the threshold corresponding with significant association between gene expression and individual of origin. X-axis was truncated at 8.0; 0.67% of the data are not plotted here for visualization purposes. Calculations include data from all lines. B. Density plot of total variance in LCLs and iPSCs. X-axis was truncated at 0.1; 4.0% of the data are not plotted here. Calculations include data from all lines. C. Density plot of between donor variance to within donor variance in gene expression for all expressed genes in iPSCs and LCLs. The dotted line indicates the threshold corresponding with significant association between gene expression and individual of origin. Calculations include data from all lines and all data are plotted. D. Density plot of total variance in LCLs and iPSCs. Calculations include data from all lines and all data are plotted. (TIF) [file pgen.1005216.s008.tif]

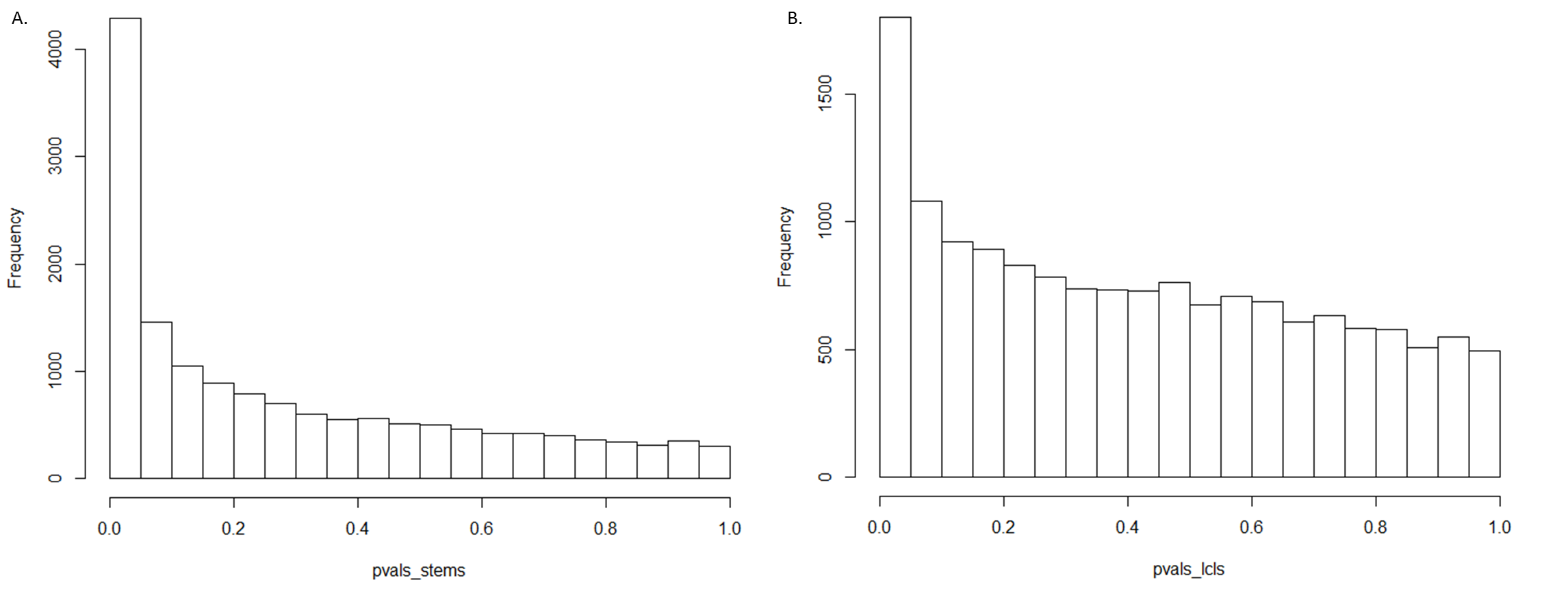

Supplement: S9 Fig — Histogram of unadjusted p-values from ANOVA F-test across the factor individual of origin for A. iPSCs and B. LCLs. Includes data from all lines. (TIF) [file pgen.1005216.s009.tif]

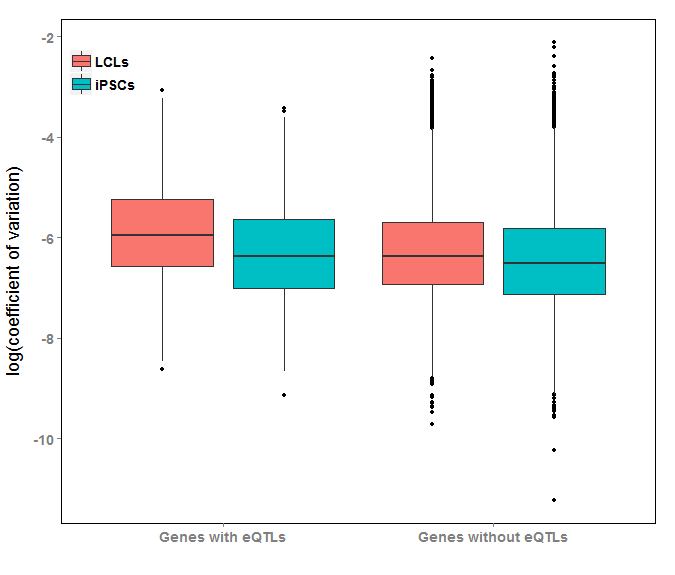

Supplement: S10 Fig — Boxplot of coefficients of variation of gene expression in genes with and without eQTLs previously identified in LCLs plotted for LCLs (P < 10-10) and iPSCs (P = 0.01). Includes data from all lines. (TIFF) [file pgen.1005216.s010.tiff]

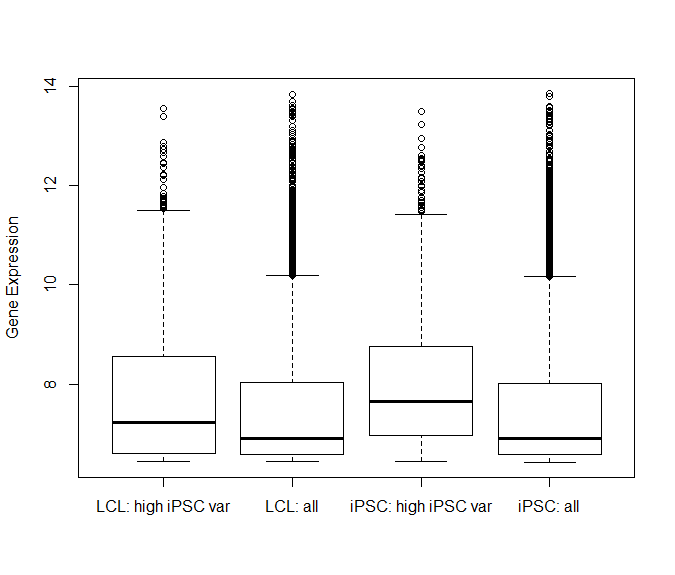

Supplement: S11 Fig — Mean gene expression levels for genes for which a donor effect was detected in iPSCs compared to all genes. Genes with a strong iPSC donor effect are expressed in LCLs, in fact with a higher mean expression value than the genome-wide average (P < 10-15). These genes exhibit significantly higher expression than the genome-wide average in iPSCs as well (P < 10-15). (TIFF) [file pgen.1005216.s011.tiff]
